# Tianma Gouteng Yin as Adjunctive Treatment for Essential Hypertension: A Systematic Review of Randomized Controlled Trials

**DOI:** 10.1155/2013/706125

**Published:** 2013-04-24

**Authors:** Jie Wang, Bo Feng, Xiaochen Yang, Wei Liu, Yongmei Liu, Yun Zhang, Gui Yu, Shengjie Li, Yuqing Zhang, Xingjiang Xiong

**Affiliations:** ^1^Department of Cardiology, Guang'anmen Hospital, China Academy of Chinese Medical Sciences, Beijing 100053, China; ^2^School of Life Sciences, Tsinghua University, Beijing 100084, China; ^3^Department of Clinical Epidemiology and Biostatistics, McMaster University, Hamilton, ON, Canada L8S 4L8

## Abstract

*Background*. Tianma Gouteng Yin (TGY) is widely used for essential hypertension (EH) as adjunctive treatment. Many randomized clinical trials (RCTs) of TGY for EH have been published. However, it has not been evaluated to justify their clinical use and recommendation based on TCM zheng classification. *Objectives*. To assess the current clinical evidence of TGY as adjunctive treatment for EH with liver yang hyperactivity syndrome (LYHS) and liver-kidney yin deficiency syndrome (LKYDS). *Search Strategy*. 7 electronic databases were searched until November 20, 2012. *Inclusion Criteria*. RCTs testing TGY combined with antihypertensive drugs versus antihypertensive drugs were included. *Data Extraction and Analyses*. Study selection, data extraction, quality assessment, and data analyses were conducted according to the Cochrane standards. *Results*. 22 RCTs were included. Methodological quality was generally low. Except diuretics treatment group, blood pressure was improved in the other 5 subgroups; zheng was improved in angiotensin converting enzyme inhibitors (ACEIs), calcium channel blockers (CCBs), and “CCB + ACEI” treatment groups. The safety of TGY is still uncertain. *Conclusions*. No confirmed conclusion about the effectiveness and safety of TGY as adjunctive treatment for EH with LYHS and LKYDS could be made. More rigorous trials are needed to confirm the results.

## 1. Introduction

Cardiovascular disease (CVD) is the leading cause of death worldwide [1, 2]. High blood pressure (BP) is the major independent risk factors for CVD [3–5]. Therefore, hypertension and blood-pressure-related disease have become an emerging epidemic and important worldwide public-health challenge [6–12]. Oral antihypertensive drugs are a milestone in the therapy of hypertension and other CVDs. However, the control rates of hypertension are still far from optimal currently [13, 14]. What is more, although treated with intensive medication, uncontrolled hypertension related symptoms (including headache, dizziness, and fatigue) and the side effects of antihypertensive drug therapy (including headache, flushing, dry cough, edema, and sexual dysfunction) are still the major problems confronting modern medicine [15–18]. Effective treatment of hypertension is limited by availability, cost, and adverse effects of conventional western medicine treatment. Thus, a certain proportion of the population has turned to complementary and alternative medicine (CAM), including traditional Chinese medicine (TCM) [19–23], for lowing BP and improving its related symptoms worldwide [24, 25].

Within Asia, TCM is widely used about 3,000 years ago [26–28]. It has formed a unique theoretical system and control methods [29–31]. TCM zheng (also called as syndrome or zheng differentiation or pattern classification) is the basic unit and the key concept in TCM theory [32–35]. Currently combination of zheng classification and biomedical diagnosis has become a hot topic both in diagnostics and treatment for the basic and clinical research [36–39]. Recently, increasing number of clinical trials and systematic reviews (SRs) showed that, as compared to antihypertensive therapy alone, Chinese herbal formula combined with antihypertensive drugs (also known as combination therapy) appear to be more effective in improving BP and symptoms in hypertensive patients with certain syndrome [40–43]. There is no doubt that, with more and more rigorous research evidences of effectiveness and safety about combination therapy, clinical studies linking TCM zheng classification and biomedicine diagnosis will lead to personalized therapeutic strategies and innovation in medical sciences [44, 45].

According to TCM theory, liver yang hyperactivity syndrome (LYHS) and liver-kidney yin deficiency syndrome (LKYDS) are the most important and common TCM zheng of essential hypertension (EH) [46]. LYHS is characterized by vertigo, tinnitus, headache, flushing, red eyes, irritability, insomnia, lassitude in lion and legs, bitter mouth, red tongue, and wiry pulse. LKYDS is always characterized by dizziness, tinnitus, headache, low fever, flushing, burning sensation of five centres, hypochondriac pain, hypopsia, lassitude in lion and legs, red tongue with scanty coating, and wiry-rapid pulse. It is worth noting that LYHS is usually caused by LKYDS. And these syndromes often appear together in EH. Molecular mechanism of LYHS and LKYDS in EH may be related to the hyperexpression of tyrosine hydroxylase (TH), and fifteen compounds of the structure and metabolic pathways mainly including amino acids, free fatty acids, and sphingosine by high performance liquid chromatography coupled with time of flight mass spectrometry (HPLC-TOFMS) [47–49]. Our previous studies showed that LYHS and LKYDS are the most important pathogenesis of EH in TCM, which could be well treated by Tianma Gouteng Yin (TGY) [46]. TGY, containing eleven commonly used herbs (*Gastrodia elata, Uncaria, *abalone shell*, Eucommia ulmoides *Oliv, achyranthes root*, Loranthus parasiticus, Gardenia, Scutellaria baicalensis* Georgi, *Leonurus japonicus, Poria cocos,* and caulis polygoni multiflori), is a classical Chinese herbal formula noted in *Za Bing Zheng Zhi Xin Yi *(*New Meanings in Syndrome and Therapy of Miscellaneous Diseases*). It has been widely used to treat hypertension-related symptoms and signs in clinical practice for decades [46]. The most common symptoms include headache, dizziness, insomnia, and lassitude in lion and legs, red tongue with scanty coating, and wiry-rapid pulse, which belong to LYHS and LKYDS in TCM [46]. The mechanism of the formula may be suppressing liver yang hyperactivity and nourishing the liver and kidney. Recently, modern researches showed that TGY could not only improve hypertension-related symptoms and signs, but also lower BP in vitro and in vivo [50–60]. Biochemically, TGY appears to have good effect in inhibiting vascular smooth muscle cell proliferation and promoting apoptosis by decreasing TGF-*β*1 and Bcl-2, improving vascular remodeling, inhibiting left ventricular hypertrophy and myocardial fibrosis, regulating rennin-angiotensin system (RAS) and Ca^2+^ overload in vascular smooth muscle cells, improving SOD, nitric oxide and insulin resistance, and decreasing endothelin, MDA, and IGF-1, so as to lower the arterial pressure [50–60]. 

Currently, a number of clinical trials have been published in Chinese language by TGY used alone or combined with antihypertensive drugs for EH. However, there is no critically appraised evidence such as SRs or meta-analyses to assess clinical efficacy and safety of TGY combined with antihypertensive drugs as an adjunct therapy for hypertensive patients based on TCM zheng classification. The PAPER aims to evaluate the beneficial and harmful effects of TGY for EH in randomized trials. To our knowledge, this is the first zheng classification-based systematic English review on TGY for EH.

## 2. Methods

### 2.1. Database and Search Strategies

Literature searches were conducted in the Cochrane Library (July, 2012), PubMed, EMBASE, Chinese National Knowledge Infrastructure (CNKI), Chinese Scientific Journal Database (VIP), Chinese Biomedical Literature Database (CBM), and Wanfang data. The reference list of retrieved papers was also searched. Since TGY was mainly used and researched in China, four main databases in Chinese were searched to retrieve the maximum possible number of trials of TGY combined with antihypertensive drugs for EH. All of those searches ended on November 20, 2012. Ongoing registered clinical trials were searched in the website of Chinese clinical trial registry (http://www.chictr.org/en/) and international clinical trial registry by US national institutes of health (http://clinicaltrials.gov/). The following search terms were used individually or combined: “Tianma Gouteng yin,” “Tianma Gouteng decoction,” “Tianma Gouteng tang,” “hypertension,” “essential hypertension,” “clinical trial,” and “randomized controlled trial.” The bibliographies of included studies were searched for additional references. 

### 2.2. Inclusion Criteria

All the parallel randomized controlled trials (RCTs) of all the prescriptions based on “Tianma Gouteng yin” combined with antihypertensive drugs compared to antihypertensive drugs for EH with LYHS and LKYDS were included. According to the principle of the similarity of TCM formula [42, 61], the number of modified herbs should not be more than 5, so that to ensure the similarity is greater than or equal to 0.5. And the key herbs in the modified Tianma Gouteng yin should include *Gastrodia elata*, *Uncaria*, and abalone shell, according to the theory of TCM. There were no restrictions on population characteristics, language and publication type. Duplicated publications reporting the same groups of participants were excluded.

### 2.3. Data Extraction and Quality Assessment

Two authors conducted the literature searching (X. J. Xiong, W. Liu), study selection (X. J. Xiong, B. Feng), and data extraction (X. J. Xiong, X. C. Yang) independently. The extracted data included authors, title of study, year of publication, study size, age and sex of the participants, details of methodological information, name and component of Chinese herbs, treatment process, details of the control interventions, outcomes, and adverse effects for each study. Disagreement was resolved by discussion and reached consensus through a third party (J. Wang). 

Criteria from the Cochrane Handbook for Systematic Review of Interventions, Version 5.1.0 were used to assess the methodological quality of trials (X. J. Xiong, Y. Zhang) [62]. The items included random sequence generation (selection bias), allocation concealment (selection bias), blinding of participants and personnel (performance bias), blinding of outcome assessment (detection bias), incomplete outcome data (attrition bias), selective reporting (reporting bias), and other bias. The quality of all the included trials was categorized to low/unclear/high risk of bias (“Yes” for a low of bias, “No” for a high risk of bias, “Unclear” otherwise). Then trials were categorized into three levels: low risk of bias (all the items were in low risk of bias), high risk of bias (at least one item was in high risk of bias), unclear risk of bias (at least one item was in unclear risk of bias). 

### 2.4. Data Synthesis

Revman 5.1 software provided by the Cochrane Collaboration was used for data analyses. Dichotomous data were presented as risk ratio (RR) and continuous outcomes as mean difference (MD), both with 95% confidence interval (CI). Heterogeneity was recognized significant when *I* ^2^ ≥ 50%. Fixed effects model was used if there is no significant heterogeneity of the data; random effects model was used if significant heterogeneity existed (50% < *I* ^2^ < 85%). Publication bias would be explored by funnel plot analysis if sufficient studies were found. 

## 3. Result

### 3.1. Description of Included Trials

**Figure 1:**
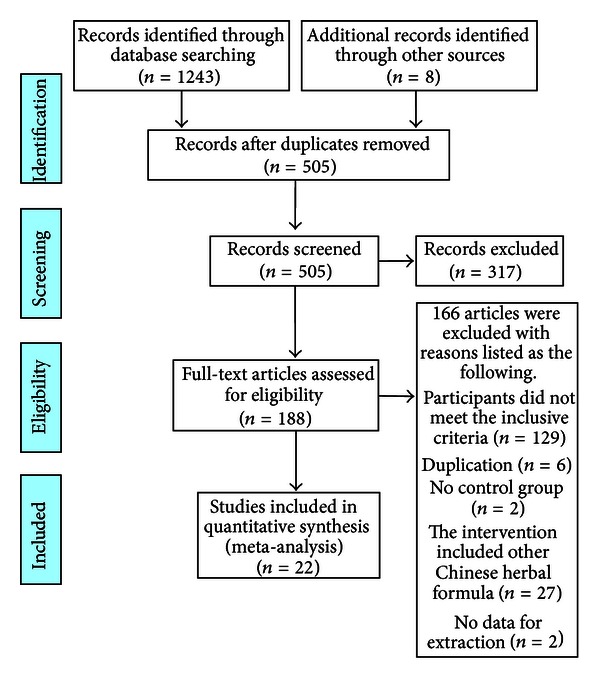
PRISMA 2009 flow diagram.

A flow chart depicted the search process and study selection (as shown in Figure 1). After primary searches from the seven databases both in Chinese and English, 1243 articles were retrieved: Cochrane Library (*n* = 1), Pubmed (*n* = 6), EMBASE (*n* = 19), CNKI (*n* = 499), VIP (*n* = 247), CBM (*n* = 270), and Wanfang data (*n* = 201). 505 articles were screened after 746 duplicates were removed. After reading the titles and abstracts, 317 articles of them were excluded. Full texts of 188 articles were retrieved, and 166 articles were excluded with reasons listed as the following: participants did not meet the inclusive criteria (*n* = 129), duplication (*n* = 6), no control group (*n* = 2), the intervention included other Chinese herbal formula (*n* = 27), and no data for extraction (*n* = 2). In the end, 22 RCTs [63–84] were included. All the RCTs were conducted in China and published in Chinese. The characteristics of included trials were listed in Table 1.

1808 patients with EH were included. There was a wide variation in the age of subjects (30–74 years). Twenty-two included clinical trials specified four diagnostic criteria of hypertension, eighteen trials [63–67, 69–74, 76–78, 80, 81, 83, 84] used 1999 WHO-ISH guidelines for the management of hypertension (1999 WHO-ISH GMH), two trials [75, 79] used Chinese Guidelines for the Management of Hypertension-2005 (CGMH-2005), one trial [82] used Chinese Guidelines for the Management of Hypertension-1999 (CGMH-1999), and one trial [68] only demonstrated patients with EH without detailed information. Twenty-two (22) trials specified three diagnostic criteria of LYHS and LKYDS in TCM, seventeen trials [64, 66–70, 72–75, 78–84] used Guidelines of Clinical Research of New Drugs of Traditional Chinese Medicine (GCRNDTCM), two trials [71, 76] used Chinese internal medicine (CIM), and three trials [63, 65, 77] only demonstrated patients with LYHS and LKYDS without detailed information about TCM diagnostic criteria.

Interventions included all the prescriptions based on “Tianma Gouteng yin” combined with antihypertensive drugs. The controls included antihypertensive drugs alone. The total treatment duration ranged from 2 to 12 weeks. The variable prescriptions are presented in Table 1. The compositions of Chinese herbal formula Tianma Gouteng yin are presented in Table 2. All of the 22 trials used the BP, including systolic blood pressure (SBP) and diastolic blood pressure (DBP), as the outcome measure. 9 trials [63, 69, 71–75, 78, 81] used TCM zheng to evaluate treatment effects as the other outcome measure.

### 3.2. Methodological Quality of Included Trials

The majority of the included clinical trials were assessed to be of general poor methodological quality according to the predefined quality assessment criteria. The randomized allocation of participants was mentioned in all trials; however, only seven trials have described the specific methods for sequence generation including random number table [64, 75, 80, 84] and drawing [74, 81]. One trial [67] only reported simple random sampling without concrete information. The remaining fifteen studies [63, 65, 66, 68–73, 76–79, 82, 83] did not mention the random sequence generation. There is also no sufficient information provided to judge whether it was conducted properly or not. Allocation concealment was not reported in all the trials. Only one trial [67] used the blinding of participants and personnel; however, no trial reported blinding of outcome assessment. Three trials [67–69] reported drop-out or withdraw. A pretrial estimation of sample size and followup had not been reported in all of included trials. We have tried to contact with authors by telephone, email, and other ways for further information about the trials; however, no information could be provided until now. The results of the assessment of risk of bias are presented in Table 3.

### 3.3. Effect of the Interventions

All the included trials [63–84] compared TGY combined with antihypertensive drugs with antihypertensive drugs alone. A change in BP was reported in all the trials. According to the different control drugs, it could be divided into six subgroups. 

#### 3.3.1. TGY Plus ACEI versus ACEI

13 trials compared TGY plus angiotensin converting enzyme inhibitors (ACEIs) versus ACEI (including captopril, enalapril, and perindopril) [63, 65, 67, 69–71, 73, 74, 80–84].

*Blood Pressure (BP). *8 trial [65, 71, 73, 74, 80–83] used three classes to evaluate treatment effects on BP: significant effective (DBP decreased by 10 mmHg reaching the normal range, or DBP has not yet returned to normal, but has been reduced ≥20 mmHg), effective (DBP decreased to less than 10 mmHg reaching the normal range, or DBP decreased by 10–19 mmHg, but did not reach the normal range, or, SBP decreased ≥30 mmHg), and ineffective (Not to meet the above standards). The trial showed significant difference between treatment and control group (RR: 3.16 [1.94, 5.14]; *P* < 0.00001) (Table 4).

When it comes to SBP, 5 independent trials [63, 67, 69, 70, 84] showed significant heterogeneity in the results, chi-square = 20.01, (*P* = 0.0005); *I* ^2^ = 80%. Thus, random-effects model was used for statistical analysis. The meta-analysis showed that there are significant beneficial effect on the combination group compare to captopril group (WMD: −6.71 [−6.94, −6.49]; *P* < 0.00001) (Table 5). 

When it comes to DBP, 5 independent trials [63, 67, 69, 70, 84] showed significant heterogeneity in the results, chi-square = 14.66, (*P* = 0.005); *I* ^2^ = 73%. Thus, random-effects model was used for statistical analysis. The meta-analysis showed there are significant beneficial effects on the combination group compared to captopril group (WMD: −4.60 [−5.11, −4.09]; *P* < 0.00001) (Table 6).

*TCM Zheng.* 6 trials [63, 69, 71, 73, 74, 81] used three classes to measure TCM zheng (just LYHS and LKYDS): significant effective (The main symptoms such as headache, dizziness, palpitations, insomnia, tinnitus, and irritability disappear, or TCM zheng scores reduced rate ≥70%), effective (The main symptoms relieved, or, 70% > TCM zheng scores reduced rate ≥30%), and ineffective (The main symptoms do not change, or TCM zheng scores reduced rate <30%). Meta-analysis showed significant difference in favor of the combination group compared to ACEI group (RR: 6.15 [3.38, 11.18]; *P* < 0.00001) (Table 7).

#### 3.3.2. TGY Plus CCB versus CCB

5 trials compared TGY plus calcium channel blockers (CCB) versus CCB (including nifedipine sustained release tablets, amlodipine besylate, nifedipine, and levamlodipine) [64, 72, 76–78].

*Blood Pressure (BP).* 2 trials [77, 78] used three classes to evaluate treatment effects on BP. The trials showed significant difference between treatment and control group (RR: 8.97 [2.59, 31.04]; *P* = 0.0005) (Table 4). 

When it comes to SBP, 3 independent trials [64, 72, 76] showed significant heterogeneity in the results, chi-square = 10.10, (*P* = 0.006); *I* ^2^ = 80%. Thus, random-effects model was used for statistical analysis. The meta-analysis showed there are significant beneficial effects on the combination group compared to captopril group (WMD: −12.03 [−13.52, −10.54]; *P* < 0.00001) (Table 5). 

When it comes to DBP, 3 independent trials [64, 72, 76] showed significant heterogeneity in the results, chi-square = 12.39, (*P* = 0.002); *I* ^2^ = 84%. Thus, random-effects model was used for statistical analysis. The meta-analysis showed there are significant beneficial effects on the combination group compared to captopril group (WMD: −7.98 [−8.85, −7.12]; *P* < 0.00001) (Table 6).

*TCM Zheng.* 2 trials [72, 78] used three classes to evaluate treatment effects on TCM zheng. The trials showed significant difference between treatment and control group (RR: 5.56 [2.50, 12.37]; *P* < 0.0001) (Table 7).

#### 3.3.3. TGY Plus Diuretics versus Diuretics

1 trial compared TGY plus diuretics versus diuretics (including hydrochlorothiazide) [68]. When it comes to SBP, it [68] showed no applicable heterogeneity in the result. Thus, fixed-effects model was used for statistical analysis. The meta-analysis showed there is no significant beneficial effect on the combination group compared to hydrochlorothiazide group (WMD: 0.28 [−0.72, 1.28]; *P* = 0.58) (Table 5). 

When it comes to DBP, it [68] showed no applicable heterogeneity in the result. Thus, fixed-effects model was used for statistical analysis. The meta-analysis showed there is no significant beneficial effect on the combination group compared to hydrochlorothiazide group (WMD: 0.44 [−0.52, 1.40]; *P* = 0.37) (Table 6).

#### 3.3.4. TGY Plus ARB versus ARB

1 trial compared TGY plus angiotensin II receptor blockers (ARB) versus ARB (including telmisartan) [79]. When it comes to SBP, it showed no applicable heterogeneity in the result. Thus, fixed-effects model was used for statistical analysis. The meta-analysis showed there is significant beneficial effect on the combination group compared to telmisartan group (WMD: −3.70 [−3.78, −3.62]; *P* < 0.00001) (Table 5). 

When it comes to DBP, it showed no applicable heterogeneity in the result. Thus, fixed-effects model was used for statistical analysis. The meta-analysis showed there is significant beneficial effect on the combination group compared to telmisartan group (WMD: −0.80 [−1.57, −0.03]; *P* = 0.04) (Table 6).

#### 3.3.5. TGY Plus “CCB + ACEI” versus “CCB + ACEI”

1 trial [75] compared TGY plus “CCB + ACEI” versus “CCB + ACEI” (including amlodipine besylate and lisinopril).

*Blood Pressure (BP).* When it comes to SBP, it showed no applicable heterogeneity in the result. Thus, fixed-effects model was used for statistical analysis. Meta-analysis showed there is significant beneficial effect on the combination group compared to “amlodipine besylate and lisinopril” group (WMD: −11.03 [−12.72, −9.34]; *P* < 0.00001) (Table 5). 

When it comes to DBP, it showed no applicable heterogeneity in the result. Thus, fixed-effects model was used for statistical analysis. Meta-analysis showed there is significant beneficial effect on the combination group compared to “amlodipine besylate and lisinopril” group (WMD: −6.87 [−7.60, −6.14]; *P* < 0.00001) (Table 6).

*TCM Zheng.* 1 trial [75] used three classes to evaluate treatment effects on TCM zheng. It showed significant difference between treatment and control group (RR: 10.55 [1.23, 90.66]; *P* = 0.03) (Table 7).

#### 3.3.6. TGY Plus Antihypertensive Drugs versus Antihypertensive Drugs

1 trial [66] compared TGY plus antihypertensive drugs versus antihypertensive drugs with no detailed information. It used three classes to evaluate treatment effects on BP. The trial showed significant difference between combination group and antihypertensive drugs group (RR: 3.53 [0.63, 19.83]; *P* = 0.01) (Table 4).

### 3.4. Publication Bias

The number of trials was too small to conduct any sufficient additional analysis of publication bias.

### 3.5. Adverse Effect

Eight out of twenty-two trials mentioned the adverse effect [67, 68, 70, 74, 76, 80, 81, 84]. Eight trials reported five specific symptoms including cough, vomiting, flushing, rash, and edema. Among them, no adverse events were found in one trial [80]. Two trials reported cough and vomiting in combination group and antihypertensive drugs group, respectively [67, 68]. Three trials reported cough in both TGY plus captopril group and captopril group [70, 74, 81]. One trial mentioned flushing in both TGY plus nifedipine sustained release tablets group and nifedipine sustained release tablets group [76]. One trial mentioned cough, rash, and edema in TGY plus captopril group, and cough and rash in captopril group [84]. 

## 4. Discussion

Due to the potential side effects of antihypertensive drugs, natural herbal products have been favored by people all over the world. Chinese herbal formula has made great contributions to the health and well-being of the people for their unique advantages in preventing and curing diseases, rehabilitation and health care. Currently, with increasing popularity of TCM [85, 86], more and more systematic reviews (SRs) and meta-analysis have been conducted to assess the efficiency of TCM for EH [87–93]. It is demonstrated that Chinese herbal medicine could not only contribute to low BP smoothly, recover the circadian rhythm of BP, but also improve symptoms and TCM zheng especially [94]. The health-enhancing qualities of TGY, a classical Chinese formula, have been dispensed and used in China for many years. As an adjunctive treatment to antihypertensive drugs, TGY is a popular classical TCM formula for EH. And until now, more and more researches about TGY have been conducted in China including 2 SRs [95, 96]. Among them, one is published in English in Cochrane library [95], and the other is in Chinese [96]. However, due to different search strategies and databases both in English and Chinese, no trial was got in this Cochrane Database of Systematic Reviews, and thus no conclusions can be made about the role of TGY in the treatment of EH [95]. Another one compared TGY plus enalapril with enalapril with positive conclusion [96]. However, as a famous adjunctive treatment in EH, the role of TGY is still unclear. This study aims to assess the current clinical evidence of TGY for EH with LYHS and LKYDS based on TCM zheng classification.

This systematic review included 22 randomized trials and a total of 1808 participants. As compared to ACEI, positive results in SBP (WMD: −6.71 [−6.94, −6.49]; *P* < 0.00001), DBP (WMD: −4.60 [−5.11, −4.09]; *P* < 0.00001), BP (RR: 3.16 [1.94, 5.14]; *P* < 0.00001), and TCM zheng (RR: 6.15 [3.38, 11.18]; *P* < 0.00001) were found about TGY plus ACEI, indicating that SBP and DBP could be decreased by 6.71 mmHg and 4.60 mmHg, respectively, and TCM zheng (just LYHS and LKYDS) could be improved after the combination therapy. As compared to CCB, positive results in SBP (WMD: −12.25 [−13.52, −10.54]; *P* < 0.00001), DBP (WMD: −7.98 [−8.85, −7.12]; *P* < 0.00001), BP (RR: 8.97 [2.59, 31.04]; *P* = 0.0005), and TCM zheng (RR: 5.56 [2.50, 12.37]; *P* < 0.0001) were found about TGY plus CCB, indicating that SBP and DBP could be decreased by 12.25 mmHg and 7.98 mmHg, respectively, and TCM zheng could be improved after the combination therapy. As compared to diuretics, there is no difference between TGY plus diuretics and diuretics in SBP (WMD: 0.28 [−0.72, 1.28]; *P* = 0.58) and DBP (WMD: 0.44 [−0.52, 1.40]; *P* = 0.37), indicating that no more beneficial effect was found in the combination therapy. As compared to ARB, positive results in SBP (WMD: −3.70 [−3.78, −3.62]; *P* < 0.00001) and DBP (WMD: −0.80 [−1.57, −0.03]; *P* = 0.04) were found about TGY plus ARB, indicating that SBP and DBP could be decreased by 3.70 mmHg and 0.80 mmHg, respectively, after the combination therapy. As compared to “CCB + ACEI,” positive results in SBP (WMD: −11.03 [−12.72, −9.34]; *P* < 0.00001), DBP (WMD: −6.87 [−7.60, −6.14]; *P* < 0.00001), and TCM zheng (RR: 10.55 [1.23, 90.66]; *P* = 0.03) were found about TGY plus “CCB + ACEI,” indicating that SBP and DBP could be decreased by 11.03 mmHg and 6.87 mmHg, respectively, and TCM zheng could be improved after the combination therapy. As compared to antihypertensive drugs, positive results in BP (RR: 3.53 [0.63, 19.83]; *P* = 0.01) was found about TGY plus antihypertensive drugs, indicating that BP could be improved after the combination therapy. In conclusion, except diuretics treatment group, BP was improved in the other 5 subgroups; TCM zheng was improved in ACEI, CCB, and “CCB + ACEI” treatment groups. However, although the meta-analysis showed positive results, no confirmed conclusion about the effectiveness and safety of TGY as adjunctive treatment for EH could be made according to current evidence due to the small sample size, poor methodological qualities, and significant heterogeneity of included trials. 

The following limitations of this review should be considered. Firstly, according to the predefined evaluation criteria, the quality of methodology is generally low in most of the included trials. There is insufficient reporting of generation methods of the allocation sequence and allocation concealment, which might lead to potential selection bias. Although it is claimed that patients were randomly assigned into two groups, we are still unable to judge if it is conducted really. Most of clinical trials have not implemented double blind in this review, and only one trial used blinding of participants and personnel, which might lead to potential performance bias and detection bias. Therefore, assessment of outcomes was prone to significant systemic errors. Only 1 trial out of the 22 included studies has placebo control. T. J. Kaptchuk once pointed out that, in alternative medicine, the main question regarding placebo has been whether a given therapy has more than a placebo effect [97]. It also regarded that only prospective trials directly comparing the placebo effects of unconventional and mainstream medicine can provide reliable evidence to support such claims [97, 98]. In this review, the result would be positive because of nonspecific placebo effects. In our opinion, the reason why placebo is not implemented may be related to the characteristics of Chinese herbs including appearance, taste, and smell [99]. Drop-out was only reported in 3 trials, and most of the trials havenot reported intention to treat analysis in details. Thus, results generated from these trials should be interpreted with caution. Additionally, most of the trials were small sample size and single-center. None of the trials have reported the sample size estimation, which placed the statistical analysis's validity in doubt.

Secondly, only 8 out of 22 trials mentioned the adverse effects, and most of the trials havenot realized the importance of adverse effects of either Chinese herbal formula or herb-drug interaction. Herb-drug interaction, just botanical and chemical drugs, is an emerging issue of integrative medicine [100–104]. It has raised more and more concern. In this review, almost all the reported adverse events such as cough, vomiting, flushing, rash, and edema in combination group may partially be related to the adverse events of antihypertensive drugs. Thus, a definite conclusion about the safety of TGY combined with antihypertensive drugs still cannot be drawn. 

Thirdly, zheng is a unique concept in TCM [105, 106]. It is identified from a comprehensive analysis of clinical information by TCM practitioner from four main diagnostic TCM methods: observation, listening, questioning, and pulse analyses [107]. In other words, zheng classification is a traditional diagnostic method to categorize patients based on their different conditions. All diagnostic and therapeutic methods in TCM are based on the differentiation of TCM syndrome, and this concept has been used for thousands of years in China [108]. Clinical trials based on TCM zheng classification may lead to more valid conclusions about Chinese herbal formula [109–114]. In our review, TGY combined with antihypertensive drugs appeared to be more effective than antihypertensive drugs alone for LYHS and LKYDS in EH. However, 3 trials did not provide detailed information about TCM diagnostic criteria, and only 9 trials used TCM zheng to evaluate treatment effects as the other outcome measure. Thus, clear diagnostic criteria and objective evaluation of TCM zheng are warranted in further clinical trials. 

In conclusion, this is the first zheng classification-based meta-analysis of randomized, controlled trials to assess the efficacy and safety of TGY as adjunctive treatment in patients with EH. However, current clinical evidence of TGY for EH with LYHS and LKYDS was so weak. More rigorous trials with high quality are needed to generate high level of evidence and confirm the results.

## Figures and Tables

**Table 1:** Characteristics and methodological quality of included studies.

Study ID	Sample	Diagnosis standard	Intervention	Control	Course (week)	Outcome measure
Wang et al., 1998 [63]	98	1999 WHO-ISH GMH; TCM diagnostic criteria (unclear)	modified TGY (1 dose/d) + control	captopril (25 mg tid)	4	BP; TCM zheng
Ren and Zhao, 2012 [64]	60	1999 WHO-ISH GMH; GCRNDTCM	modified TGY (300 mL/d) + control	nifedipine sustained release tablets (10 mg bid)	6	BP
Fu, 2005 [65]	116	1999 WHO-ISH GMH; TCM diagnostic criteria (unclear)	modified TGY (250 mL/d) + control	enalapril (10 mg bid)	2	BP
Qin, 2010 [66]	45	1999 WHO-ISH GMH; GCRNDTCM	TGY (1 dose/d) + control	antihypertensive drugs (no detailed information)	4	BP
Hu et al., 2009 [67]	39	1999 WHO-ISH GMH; GCRNDTCM	TGY (1 dose/d) + captopril	captopril (12.5–25 mg bid) + TGY placebo	4	BP
Sun and Huang, 2007 [68]	60	hypertension diagnostic criteria (unclear); GCRNDTCM	TGY (1 dose/d) + control	hydrochlorothiazide (12.5 mg bid)	4	BP
Wang et al., 2008 [69]	80	1999 WHO-ISH GMH; GCRNDTCM	TGY (1 dose/d) + control	captopril (25 mg tid)	4	BP; TCM zheng
Fang et al., 2008 [70]	60	1999 WHO-ISH GMH; GCRNDTCM	TGY (1 dose/d) + control	captopril (25 mg tid)	4	BP
Li, 2006 [71]	80	1999 WHO-ISH GMH; CIM	modified TGY (1 dose/d) + control	captopril (12.5 mg bid)	4	BP; TCM zheng
Liu, 2011 [72]	86	1999 WHO-ISH GMH; GCRNDTCM	modified TGY (1 dose/d) + control	amlodipine besylate (5 mg qd)	8	BP; TCM zheng
Liu et al., 2009 [73]	60	1999 WHO-ISH GMH; GCRNDTCM	TGY (1 dose/d) + control	perindopril (25 mg tid)	4	BP; TCM zheng
Chen, 2009 [74]	100	1999 WHO-ISH GMH; GCRNDTCM	TGY (1 dose/d) + control	captopril (25 mg tid)	6	BP; TCM zheng
Zhang and Yang, 2011 [75]	60	CGMH-2005; GCRNDTCM	modified TGY (1 dose/d) + control	amlodipine besylate (5 mg qd) + lisinopril (10 mg qd)	4	BP; TCM zheng
Liu and Li, 2011 [76]	100	1999 WHO-ISH GMH; CIM	TGY (1 dose/d) + control	nifedipine sustained release tablets (10 mg bid)	4	BP
Wei, 2012 [77]	98	1999 WHO-ISH GMH; TCM diagnostic criteria (unclear)	TGY (1 dose/d) + control	nifedipine (10 mg bid)	4	BP
Li, 2011 [78]	120	1999 WHO-ISH GMH; GCRNDTCM	TGY (1 dose/d) + control	levamlodipine (5 mg qd)	4	BP
Lin and Liu, 2010 [79]	94	CGMH-2005; GCRNDTCM	TGY (1 dose/d) + control	telmisartan (20 mg qd)	4	BP
Zhou and Zhang, 2011 [80]	72	1999 WHO-ISH GMH; GCRNDTCM	modified TGY (1 dose/d) + control	captopril (25 mg bid)	4	BP
Chen, 2008 [81]	100	1999 WHO-ISH GMH; GCRNDTCM	TGY (1 dose/d) + control	captopril (25 mg tid)	6	BP; TCM zheng
Du, 2012 [82]	100	CGMH-1999; GCRNDTCM	modified TGY (1 dose/d) + control	captopril (25 mg tid)	4	BP
Cheng, 2010 [83]	100	1999 WHO-ISH GMH; GCRNDTCM	modified TGY (1 dose/d) + control	enalapril (10 mg qd)	12	BP
Zhu, 2009 [84]	80	1999 WHO-ISH GMH; GCRNDTCM	TGY (1 dose/d) + control	captopril (25 mg tid)	4	BP

**Table 2:** Composition of formula.

Study ID	Formula	Composition of formula
Wang et al., 1998 [63]	modified TGY	*Salvia miltiorrhiza* 30 g, red peony root 20 g, *Gastrodia elata *12 g, *Uncaria* 30 g, abalone shell 30 g, *Eucommia ulmoides *Oliv 15 g, achyranthes root 30 g, *Loranthus parasiticus *15 g, *Gardenia* 12 g, *Scutellaria baicalensis* Georgi 12 g, *Leonurus japonicus *20 g, *Poria cocos *15 g, caulis polygoni multiflori 30 g

Ren and Zhao, 2012 [64]	modified TGY	*Gastrodia elata *9 g, *Uncaria* 12 g, abalone shell 18 g, *Eucommia ulmoides *Oliv 9 g, achyranthes root 12 g, *Loranthus parasiticus*9 g, *Gardenia* 9 g, *Scutellaria baicalensis* Georgi 9 g, *Leonurus japonicus *9 g, *scu*9 g, caulis polygoni multiflori 9 g

Fu, 2005 [65]	modified TGY	*Gastrodia elata *18 g, *Uncaria* 12 g, abalone shell 18 g, *Eucommia ulmoides *Oliv 9 g, achyranthes root 15 g, *Loranthus parasiticus *9 g, *Gardenia* 9 g, *Scutellaria baicalensis* Georgi 12 g, *Leonurus japonicus *9 g, *Poria cocos *9 g, caulis polygoni multiflori 9 g, *Prunella vulgaris* 12 g

Qin, 2010 [66]	TGY	*Gastrodia elata*, *Uncaria*, abalone shell, *Eucommia ulmoides *Oliv, achyranthes root, *Loranthus parasiticus*, *Gardenia*, *Scutellaria baicalensis* Georgi, *Leonurus japonicus*, *Poria cocos*, caulis polygoni multiflori

Hu et al., 2009 [67]	TGY	*Gastrodia elata *9 g, *Uncaria* 12 g, abalone shell 18 g, *Eucommia ulmoides *Oliv 9 g, achyranthes root 12 g, *Loranthus parasiticus *9 g, *Gardenia* 9 g, *Scutellaria baicalensis* Georgi 9 g, *Leonurus japonicus *9 g, *Poria cocos *9 g, caulis polygoni multiflori 9 g

Sun and Huang, 2007 [68]	TGY	*Gastrodia elata *9 g, *Uncaria* 12 g, abalone shell 18 g, *Eucommia ulmoides *Oliv 9 g, achyranthes root 12 g, *Loranthus parasiticus *9 g, *Gardenia* 9 g, *Scutellaria baicalensis* Georgi 9 g, *Leonurus japonicus *9 g, *Poria cocos *9 g, caulis polygoni multiflori 9 g

Wang et al., 2008 [69]	TGY	*Gastrodia elata *15 g, *Uncaria* 30 g, abalone shell 21 g, *Eucommia ulmoides *Oliv 12 g, achyranthes root 15 g, *Loranthus parasiticus *12 g, *Gardenia* 12 g, *Scutellaria baicalensis* Georgi 12 g, *Leonurus japonicus *12 g, *Poria cocos *12 g, caulis polygoni multiflori 12 g

Fang et al., 2008 [70]	TGY	*Gastrodia elata *9 g, *Uncaria* 12 g, abalone shell 18 g, *Eucommia ulmoides *Oliv 9 g, achyranthes root 12 g, *Loranthus parasiticus *9 g, *Gardenia* 9 g, *Scutellaria baicalensis* Georgi 9 g, *Leonurus japonicus *9 g, *Poria cocos *9 g, caulis polygoni multiflori 9 g

Li, 2006 [71]	modified TGY	*Gastrodia elata *10 g, *Uncaria* 12 g, abalone shell 30 g, achyranthes root 15 g, *Loranthus parasiticus *15 g, *Scutellaria baicalensis* Georgi 10 g, *Leonurus japonicus *20 g, caulis polygoni multiflori 25 g, white peony root 15 g, *Chrysanthemum* 12 g

Liu, 2011 [72]	modified TGY	*Gastrodia elata *10 g, *Uncaria* 15 g, abalone shell 20 g, *Eucommia ulmoides *Oliv 10 g, achyranthes root 15 g, *Loranthus parasiticus *15 g, *Gardenia* 10 g, *Scutellaria baicalensis* Georgi 10 g, *Poria cocos *30 g, caulis polygoni multiflori 15 g, *Apocynum* 10 g

Liu et al., 2009 [73]	TGY	*Gastrodia elata *9 g, *Uncaria* 12 g, abalone shell 18 g, *Eucommia ulmoides *Oliv 9 g, achyranthes root 12 g, *Loranthus parasiticus *9 g, *Gardenia* 9 g, *Scutellaria baicalensis* Georgi 9 g, *Leonurus japonicus *9 g, *Poria cocos *9 g, caulis polygoni multiflori 9 g

Chen, 2009 [74]	TGY	*Gastrodia elata* 12 g, *Uncaria* 9 g, abalone shell 30 g, *Eucommia ulmoides *Oliv 12 g, achyranthes root 15 g, *Loranthus parasiticus *15 g, *Gardenia* 9 g, *Leonurus japonicus *15 g, *Poria cocos *15 g, caulis polygoni multiflori 20 g

Zhang and Yang, 2011 [75]	modified TGY	*Gastrodia elata* 10 g, *Uncaria* 30 g, abalone shell 30 g, *Eucommia ulmoides *Oliv 10 g, achyranthes root 15 g, *Loranthus parasiticus *15 g, *Poria cocos *15 g, caulis polygoni multiflori 15 g, *Salvia miltiorrhiza* 30 g, *Prunella vulgaris* 15 g, keel 30 g, oyster 30 g, pearl shell 30 g, *Albizia julibrissin* 15 g, *Glycyrrhiza* 6 g

Liu and Li, 2011 [76]	TGY	*Gastrodia elata* 15 g, *Uncaria* 30 g, abalone shell 30 g, *Eucommia ulmoides *Oliv 15 g, achyranthes root 15 g, *Loranthus parasiticus *30 g, *Scutellaria baicalensis* Georgi 15 g, *Leonurus japonicus *15 g, *Poria cocos *20 g, caulis polygoni multiflori 15 g

Wei, 2012 [77]	TGY	*Gastrodia elata* 10 g, *Uncaria* 15 g, abalone shell 20 g, *Eucommia ulmoides *Oliv 10 g, achyranthes root 15 g, *Loranthus parasiticus *15 g, *Gardenia* 10 g, *Scutellaria baicalensis* Georgi 10 g, *Leonurus japonicus *15 g, *Poria cocos *15 g, caulis polygoni multiflori 10 g

Li, 2011 [78]	TGY	*Gastrodia elata* 10 g, *Uncaria* 15 g, abalone shell 20 g, *Eucommia ulmoides *Oliv 10 g, achyranthes root 15 g, *Loranthus parasiticus *15 g, *Gardenia* 10 g, *Scutellaria baicalensis* Georgi 10 g, *Leonurus japonicus *15 g, *Poria cocos *15 g, caulis polygoni multiflori 10 g

Lin and Liu, 2010 [79]	TGY	*Gastrodia elata* 9 g, *Uncaria* 12 g, abalone shell 30 g, *Eucommia ulmoides *Oliv 10 g, achyranthes root 12 g, *Loranthus parasiticus *12 g, *Gardenia* 6 g, *Scutellaria baicalensis* Georgi 9 g, *Leonurus japonicus *9 g, *Poria cocos *12 g, caulis polygoni multiflori 12 g

Zhou and Zhang, 2011 [80]	modified TGY	*Gastrodia elata* 12 g, *Uncaria* 10 g, abalone shell 10 g, *Eucommia ulmoides *Oliv 10 g, achyranthes root 10 g, *Loranthus parasiticus *10 g, *Gardenia* 9 g, *Scutellaria baicalensis* Georgi 9 g, *Leonurus japonicus *15 g, *Poria cocos *10 g, caulis polygoni multiflori 10 g, *Gentiana scabra* bge 15 g

Chen, 2008 [81]	TGY	*Gastrodia elata* 12 g, *Uncaria* 9 g, abalone shell 30 g, *Eucommia ulmoides *Oliv 12 g, achyranthes root 15 g, *Loranthus parasiticus *15 g, *Gardenia* 9 g, *Leonurus japonicus *15 g, *Poria cocos *15 g, caulis polygoni multiflori 20 g

Du, 2012 [82]	modified TGY	*Gastrodia elata* 15 g, *Uncaria* 20 g, abalone shell 15 g, *Eucommia ulmoides *Oliv 10 g, achyranthes root 10 g, *Loranthus parasiticus *10 g, *Gardenia* 6 g, *Scutellaria baicalensis* Georgi 8 g, *Leonurus japonicus *10 g, *Poria cocos *10 g, caulis polygoni multiflori 30 g; constipation plus rhubarb 5 g and aloe vera 10 g; liver-kidney yin deficiency plus glossy privet 10 g, wolfberry fruit 15 g, *Rehmanniae radix* 15 g, root of herbaceous peony 10 g; gan yang hua feng syndrome and dizziness plus antelope horn 3 g, pearl shell 15 g, keel 30 g and oyster 30 g; severe headache, flushed face and congested eyes and coating on the tongue yellow dry plus *Gentiana scabra* bge 15 g, *Chrysanthemum* 10 g, cortex moutan radicis 10 g

Cheng, 2010 [83]	modified TGY	*Gastrodia elata* 12 g, *Uncaria* 12 g, abalone shell 18 g, *Eucommia ulmoides *Oliv 12 g, achyranthes root 12 g, *Loranthus parasiticus *12 g, *Gardenia* 12 g, *Scutellaria baicalensis* Georgi 12 g, *Leonurus japonicus* 12 g, *Poria cocos *12 g, caulis polygoni multiflori 9 g, hawthorn 30 g, giant knotweed 10 g

Zhu, 2009 [84]	TGY	*Gastrodia elata* 9 g, *Uncaria* 12 g, *Eucommia ulmoides *Oliv 9 g, achyranthes root 12 g, *Loranthus parasiticus *9 g, *Gardenia* 9 g, *Scutellaria baicalensis* Georgi 9 g, *Leonurus japonicus *9 g, *Poria cocos *9 g, caulis polygoni multiflori 9 g

**Table 3:** Quality assessment of included randomized controlled trials.

Included trials	Random sequence generation	Allocation concealment	Blinding of participants and personnel	Blinding of outcome assessment	Incomplete outcome data	Selective reporting	Other sources of bias	Risk of bias
Wang et al., 1998 [63]	Unclear	Unclear	Unclear	Unclear	Yes	No	Unclear	High
Ren and Zhao, 2012 [64]	Table of random number	Unclear	Unclear	Unclear	Yes	No	Unclear	Unclear
Fu, 2005 [65]	Unclear	Unclear	Unclear	Unclear	Yes	No	Unclear	High
Qin, 2010 [66]	Unclear	Unclear	Unclear	Unclear	Yes	No	Unclear	High
Hu et al., 2009 [67]	Simple random sampling	Unclear	Yes	Unclear	No	No	Unclear	Unclear
Sun and Huang, 2007 [68]	Unclear	Unclear	Unclear	Unclear	No	No	Unclear	High
Wang et al., 2008 [69]	Unclear	Unclear	Unclear	Unclear	Yes	No	Unclear	High
Fang et al., 2008 [70]	Unclear	Unclear	Unclear	Unclear	No	No	Unclear	High
Li, 2006 [71]	Unclear	Unclear	Unclear	Unclear	Yes	No	Unclear	High
Liu, 2011 [72]	Unclear	Unclear	Unclear	Unclear	Yes	No	Unclear	High
Liu et al., 2009[73]	Unclear	Unclear	Unclear	Unclear	Yes	No	Unclear	High
Chen, 2009 [74]	Drawing	Unclear	Unclear	Unclear	No	No	Unclear	Unclear
Zhang and Yang, 2011 [75]	Table of random number	Unclear	Unclear	Unclear	Yes	No	Unclear	Unclear
Liu and Li, 2011 [76]	Unclear	Unclear	Unclear	Unclear	No	No	Unclear	High
Wei, 2012 [77]	Unclear	Unclear	Unclear	Unclear	Yes	No	Unclear	High
Li, 2011 [78]	Unclear	Unclear	Unclear	Unclear	Yes	No	Unclear	High
Lin and Liu, 2010 [79]	Unclear	Unclear	Unclear	Unclear	Yes	No	Unclear	High
Zhou and Zhang, 2011 [80]	Table of random number	Unclear	Unclear	Unclear	No	No	Unclear	Unclear
Chen, 2008 [81]	Drawing	Unclear	Unclear	Unclear	No	No	Unclear	Unclear
Du, 2012 [82]	Unclear	Unclear	Unclear	Unclear	Yes	No	Unclear	High
Cheng, 2010 [83]	Unclear	Unclear	Unclear	Unclear	Yes	No	Unclear	High
Zhu, 2009 [84]	Table of random number	Unclear	Unclear	Unclear	No	No	Unclear	Unclear

**Table 4:** Analyses of blood pressure.

Trials		Intervention (*n*/*N*)	Control (*n*/*N*)	RR [95% CI]	*P *Value
TGY plus ACEI versus ACEI					
modified TGY plus enalapril versus enalapril	1	56/60	44/56	3.82 [1.15, 12.66]	0.03
modified TGY plus captopril versus captopril	1	44/48	21/32	5.76 [1.64, 20.25]	0.006
TGY plus perindopril versus perindopril	1	29/30	26/30	4.46 [0.47, 42.51]	0.19
TGY plus captopril versus captopril	1	45/50	44/50	1.23 [0.35, 4.32]	0.75
modified TGY plus captopril versus captopril	1	34/36	30/36	3.40 [0.64, 18.13]	0.15
TGY plus captopril versus captopril	1	45/50	44/50	1.23 [0.35, 4.32]	0.75
modified TGY plus captopril versus captopril	1	48/50	42/50	4.57 [0.92, 22.73]	0.06
modified TGY plus enalapril versus enalapril	1	48/50	39/50	6.77 [1.42, 32.37]	0.02

Meta-Analysis	8	349/374	290/354	3.16 [1.94, 5.14]	<0.00001

TGY plus CCB versus CCB					
TGY plus nifedipine versus nifedipine	1	48/49	40/49	10.80 [1.31, 88.92]	0.03
TGY plus levamlodipine versus levamlodipine	1	58/60	47/60	8.02 [1.72, 37.33]	0.008

Meta-Analysis	2	106/109	87/109	8.97 [2.59, 31.04]	0.0005

TGY plus antihypertensive drugs versus antihypertensive drugs					
TGY plus antihypertensive drugs versus antihypertensive drugs	1	20/22	17/23	3.53 [0.63, 19.83]	0.01

Meta-Analysis	1	20/22	17/23	3.53 [0.63, 19.83]	0.01

**Table 5:** Analyses of systolic blood pressure.

Trials		MD [95% CI]	*P *Value
TGY plus ACEI versus ACEI			
modified TGY plus captopril versus captopril	1	−9.70 [−11.80, −7.60]	<0.00001
TGY plus captopril versus captopril	1	−6.20 [−7.03, −5.37]	<0.00001
TGY plus captopril versus captopril	1	−8.76 [−10.02, −7.50]	<0.00001
TGY plus captopril versus captopril	1	−5.30 [−9.88, −0.72]	0.02
TGY plus captopril versus captopril	1	−6.65 [−6.89, −6.41]	<0.00001

Meta-Analysis	5	−6.71 [−6.94, −6.49]	<0.00001

TGY plus CCB versus CCB			
modified TGY plus nifedipine sustained release tablets versus nifedipine sustained release tablets	1	−13.00 [−14.48, −11.52]	<0.00001
modified TGY plus amlodipine besylate versus amlodipine besylate	1	−12.60 [−13.69, −11.51]	<0.00001
TGY plus nifedipine sustained release tablets versus nifedipine sustained release tablets	1	−10.68 [−11.62, −9.74]	<0.00001

Meta-Analysis	3	−12.25 [−13.52, −10.54]	<0.00001

TGY plus diuretics versus diuretics			
modified TGY plus hydrochlorothiazide versus hydrochlorothiazide	1	0.28 [−0.72, 1.28]	0.58

Meta-Analysis	1	0.28 [−0.72, 1.28]	0.58

TGY plus ARB versus ARB			
TGY plus telmisartan versus telmisartan	1	−3.70 [−3.78, −3.62]	<0.00001

Meta-Analysis	1	−3.70 [−3.78, −3.62]	<0.00001

TGY plus “CCB + ACEI” versus “CCB + ACEI”			
modified TGY plus amlodipine besylate and lisinopril versus amlodipine besylate and lisinopril	1	−11.03 [−12.72, −9.34]	<0.00001

Meta-Analysis	1	−11.03 [−12.72, −9.34]	<0.00001

**Table 6:** Analyses of diastolic blood pressure.

Trials		MD [95% CI]	*P *Value
TGY plus ACEI versus ACEI			
modified TGY plus captopril versus captopril	1	−6.10 [−9.06, −3.14]	<0.0001
TGY plus captopril versus captopril	1	−5.00 [−5.89, −4.11]	<0.00001
TGY plus captopril versus captopril	1	−3.69 [−4.44, −2.94]	<0.00001
TGY plus captopril versus captopril	1	−3.30 [−7.12, 0.52]	0.09
TGY plus captopril versus captopril	1	−6.28 [−7.55, −5.01]	<0.00001

Meta-Analysis	5	−4.60 [−5.11, −4.09]	<0.00001

TGY plus CCB versus CCB			
modified TGY plus nifedipine sustained release tablets versus nifedipine sustained release tablets	1	−10.00 [−12.53, −7.47]	<0.00001
modified TGY plus amlodipine besylate versus amlodipine besylate	1	−8.20 [−8.58, −7.82]	<0.00001
TGY plus nifedipine sustained release tablets versus nifedipine sustained release tablets	1	−7.34 [−7.73, −6.95]	<0.00001

Meta-Analysis	3	−7.98 [−8.85, −7.12]	<0.00001

TGY plus diuretics versus diuretics			
modified TGY plus hydrochlorothiazide versus hydrochlorothiazide	1	0.44 [−0.52, 1.40]	0.37

Meta-Analysis	1	0.44 [−0.52, 1.40]	0.37

TGY plus ARB versus ARB			
TGY plus telmisartan versus telmisartan	1	−0.80 [−1.57, −0.03]	0.04

Meta-Analysis	1	−0.80 [−1.57, −0.03]	0.04

TGY plus “CCB + ACEI” versus “CCB + ACEI”			
modified TGY plus amlodipine besylate and lisinopril versus amlodipine besylate and lisinopril	1	−6.87 [−7.60, −6.14]	<0.00001

Meta-Analysis	1	−6.87 [−7.60, −6.14]	<0.00001

**Table 7:** Analyses of TCM zheng.

Trials		Intervention (*n*/*N*)	Control (*n*/*N*)	RR [95% CI]	*P *Value
TGY plus ACEI versus ACEI					
modified TGY plus captopril versus captopril	1	48/50	36/48	8.00 [1.68, 38.00]	0.009
TGY plus captopril versus captopril	1	38/40	28/40	8.14 [1.69, 39.22]	0.009
modified TGY plus captopril versus captopril	1	44/48	21/32	5.76 [1.64, 20.25]	0.006
TGY plus perindopril versus perindopril	1	29/30	23/30	8.83 [1.01, 76.96]	0.05
TGY plus captopril versus captopril	1	47/50	38/50	4.95 [1.30, 18.81]	0.02
TGY plus captopril versus captopril	1	47/50	39/50	4.42 [1.15, 16.97]	0.03

Meta-Analysis	6	253/268	185/250	6.15 [3.38, 11.18]	<0.00001

TGY plus CCB versus CCB					
modified TGY plus amlodipine besylate versus amlodipine besylate	1	42/46	26/40	5.65 [1.68, 19.04]	0.005
TGY plus levamlodipine versus levamlodipine	1	55/60	40/60	5.50 [1.90, 15.89]	0.002

Meta-Analysis	2	97/106	66/100	5.56 [2.50, 12.37]	<0.0001

TGY plus “CCB + ACEI” versus “CCB + ACEI”					
modified TGY plus amlodipine besylate and lisinopril versus amlodipine besylate and lisinopril	1	29/30	22/30	10.55 [1.23, 90.66]	0.03

Meta-Analysis	1	29/30	22/30	10.55 [1.23, 90.66]	0.03
